# Atmosphere injection of sea salts during large explosive submarine volcanic eruptions

**DOI:** 10.1038/s41598-023-41639-8

**Published:** 2023-09-02

**Authors:** M. Colombier, I. A. Ukstins, S. Tegtmeier, B. Scheu, S. J. Cronin, S. Thivet, J. Paredes-Mariño, C. Cimarelli, K.-U. Hess, Taaniela Kula, Folauhola H. Latu’ila, D. B. Dingwell

**Affiliations:** 1 Department of Earth and Environmental Sciences, Ludwig-Maximilians-Universität München, Munich, Germany; 2 School of Environment, University of Auckland, Auckland, New Zealand; 3 University of Saskatchewan, Institute of Space and Atmospheric Studies, Saskatoon, SK S7N 5E2 Canada; 4 Department of Earth Sciences, University of Geneva, Geneva, Switzerland; 5 Tonga Geological Services, Nuku’alofa, Tonga

**Keywords:** Volcanology, Atmospheric science

## Abstract

The 15 January 2022 submarine eruption at Hunga volcano was the most explosive volcanic eruption in 140 years. It involved exceptional magma and seawater interaction throughout the entire submarine caldera collapse. The submarine volcanic jet breached the sea surface and formed a subaerial eruptive plume that transported volcanic ash, gas, sea salts and seawater up to ~ 57 km, reaching into the mesosphere. We document high concentrations of sea salts in tephra (volcanic ash) collected shortly after deposition. We also discuss the potential climatic consequences of large-scale injection of salts into the upper atmosphere during submarine eruptions. Sodium chloride in these volcanic plumes can reach extreme concentrations, and dehalogenation of chlorides and bromides poses the risk of long-term atmospheric and weather impact. Salt content in rapidly collected tephra samples may also be used as a proxy to estimate the water:magma ratio during eruption, with implications for quantification of fragmentation efficiency in submarine breaching events. The balance between salt loading into the atmosphere versus deposition in ash aggregates is a key factor in understanding the atmospheric and climatic consequences of submarine eruptions.

## Introduction

Explosive submarine volcanic eruptions at shallow to intermediate water depths (typically < 200–500 up to 1000 m) may breach the sea surface and form subaerial eruption columns^1^. The largest of these eruptions have reached high levels of the atmosphere and stratosphere, and include the 2019 Anak Krakatau (Indonesia) and January 2022 Hunga volcano (Tonga) events^2,3^. Turbulent mixing of magma and seawater during shallow submarine eruptions drives flash-boiling and extensive salt precipitation^4^. Salt formation following interaction between lava and seawater has been described in a variety of volcanic settings including deep (> 2500 m below sea level) submarine lava flows at mid-ocean ridges^5^, ocean entry of lava flows during littoral explosions^6,7^, non-breaching submarine explosive eruptions^8^, and in Surtseyan eruptions, where some of the highest salt concentrations are found^9,10^. During seawater breaching events, subaerial eruption columns inject large volumes of tephra (mainly of ash grade < 2 mm diameter particles), as well as aerosols of volcanic gas, salts and seawater steam into the atmosphere with significant meteorological, hydrological and environmental implications.

During the peak climactic phase of the 2022 Hunga volcano eruption, the upper part of the plume reached 57 km^3^ injecting aerosols into the mesosphere. The impact of these aerosols and salts at such high atmospheric levels is not well known, but postulated effects include ozone destruction^11^, radiative forcing and climate warming^12^, variations in mesospheric clouds^3^, as well as impacts on regional and global climate^13^. The Hunga eruption provides a unique opportunity for understanding the production of marine-sourced salts during submarine eruptions as well as ion scavenging in the eruption column. Quantification of these processes, in turn, can inform models of atmospheric salt loading and its consequences. Here, we present data on the speciation and concentration of salts found as precipitates on the 15 January ash and discuss their influence on atmospheric processes.

## Results

**Figure 1:**
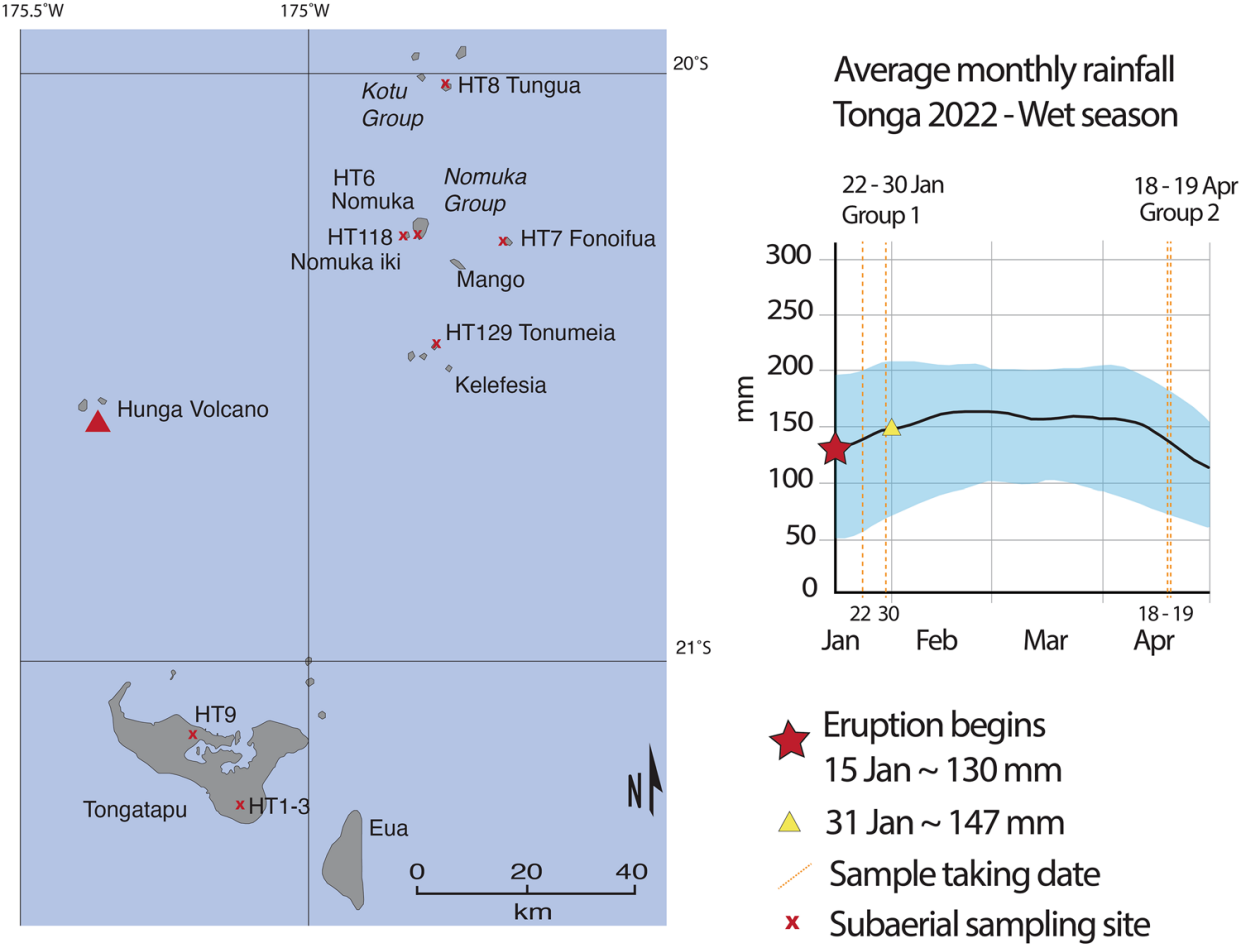
(**a**) Map showing the sampling location. (**b**) Average monthly rainfall in Tonga, for the first four-month period of 2022, modified from^40^. The solid line represents the cumulative average rainfall, shadowed area represents the 25^th^ to 75^th^ percentile bands. The date on which samples were taken is indicated so the effects of rain on the deposits can be assessed against the results.

The 15 January 2022 eruption of Hunga volcano occurred during the wet season in the Kingdom of Tonga^14^. The year 2022 exhibited a La Niña pattern and there was large variability in rainfall compared to average values. Following the eruption, there was a ~ two-week period of little to no rain. Ten samples were collected across the islands of Tonga after deposition (Fig. 1). Group 1 ash samples were collected one to two weeks after the eruption with limited exposure to rain (HT1, 3, 6, 7, 8 and 9), and Group 2 samples were collected three months later with longer environmental exposure and more rainfall interaction (HT118, 129A and 129B). Sample HT2 is from Group 1 but was washed prior to analysis and hence corresponds to a salt-free sample. Sample HT9 is the largest bulk sample and was carefully sampled by personnel from the Tonga Geoscience Services from the top of a painted shipping container on their premises in Nukualofa.

### BSE/EDX analysis

**Figure 2:**
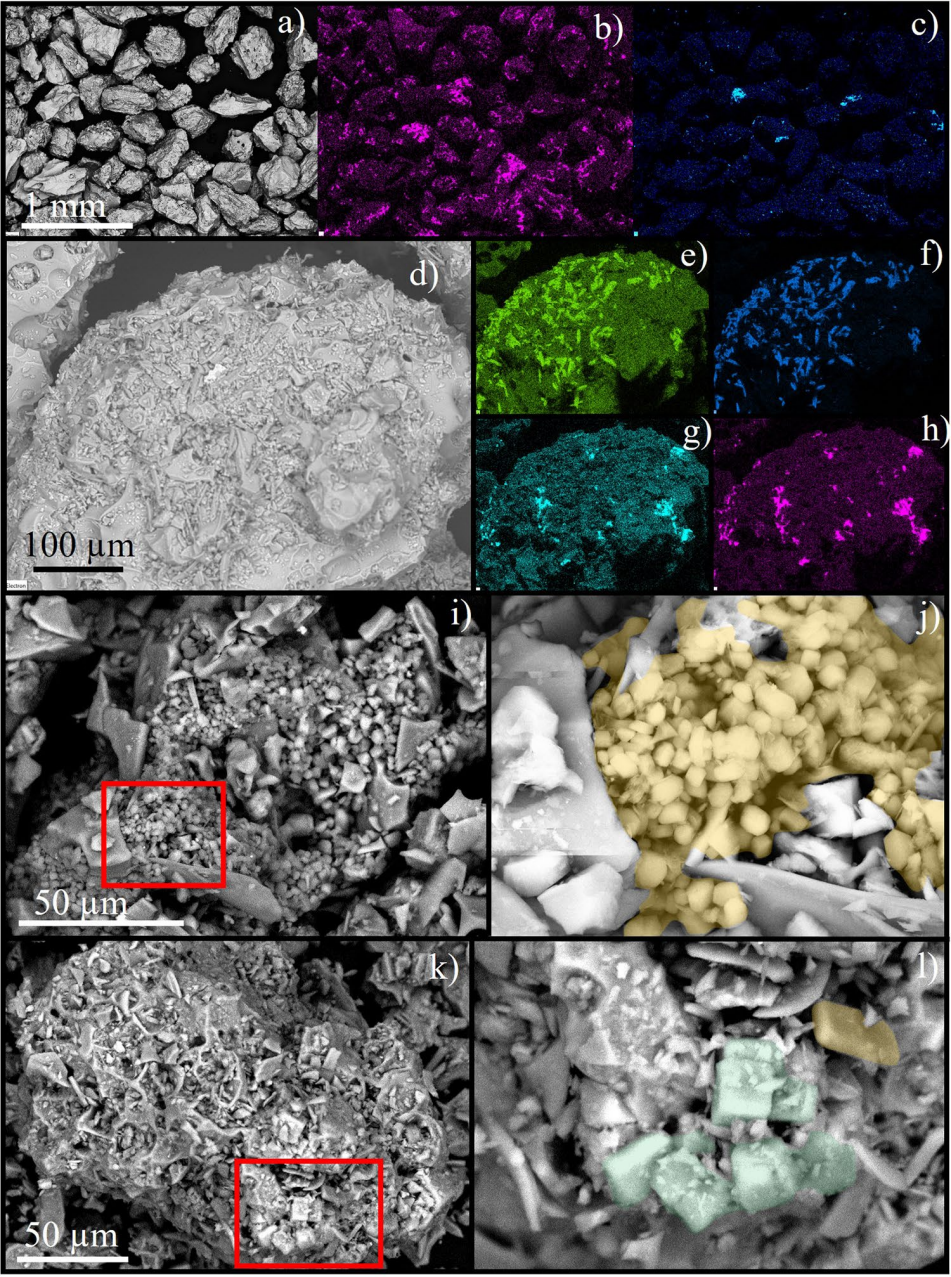
SEM analysis combining BSE images and EDX maps of Group 1 (salt-rich) samples from the 15 January 2022 eruption. (**a**–**c**) BSE image, Cl and S maps for the sample HT7 in the grain size 250–355 µm, showing that all particles are partially covered by salt phases. Note that Cl and S show a systematic correlation with Na and Ca maps (not shown here), respectively. (**d**–**h**) BSE and EDX maps for Ca-SO_4_ (**e**,**f**) and Na-Cl (**g**,**h**) to illustrate the salt coverage in the fine ash rim of a coated coarse ash particle. (**i**,**j**) Ca-sulfate cluster in sample HT6, with sulfates colourized in orange in the image (**j**). (**k**,**l**) Cluster of cubic halite crystals (colourized in green in **l**) and one Ca-SO_4_ crystal (in orange in **l**) in the fine ash rim of a coarse ash particle from sample HT1.

Salts (mostly NaCl and CaSO_4_) are present in variable concentrations in samples analysed here. The most pristine samples from Group 1 are salt-rich, compared to the later-sampled Group 2 suite. Ash particles from all deposits show evidence of coating and aggregation. Soluble salts are observed in all Group 1 samples and consist mostly of sodium chloride and calcium sulfate based on Na-Cl and Ca-S correlations in EDX maps (Fig. 2). Salts in Group 1 are almost always associated with ash particles and commonly occur in rims of fine ash surrounding coarser ash (Fig. 2). Salts are also observed as individual free crystals, which are, in turn, coated by finer ash particles. Salt coverage on grain surfaces varies greatly among particles, from 0.2% to almost 100%. Via image analysis, we estimated an average salt coverage of 7% in the fraction 710 µm–1 mm in a representative Group 1 sample (HT7), with > 80% of these salts being sodium chloride. Both chloride and sulfate are present in high number density, frequently occur as clusters, and show a range of morphologies (Fig. 2). Chloride is present as (1) euhedral cubic crystals (Fig. 2), (2) aggregates of subrounded crystals and (3) smooth coatings, possibly resulting from salt dissolution and redistribution^15^. The habit of the crystalline salts is not distinctive for Ca-sulfate. No NaCl and CaSO_4_ salts are observed in Group 2 samples, but may nevertheless be present in very low concentrations or in cavities. We also observed correlations between EDX maps of Br and Al, which suggests the presence of aluminium bromide in all samples. Finally, Fe-S phases, likely iron sulfide or iron sulphate, are also observed in both groups of samples.

### Evolved gas analysis (EGA)

**Figure 3:**
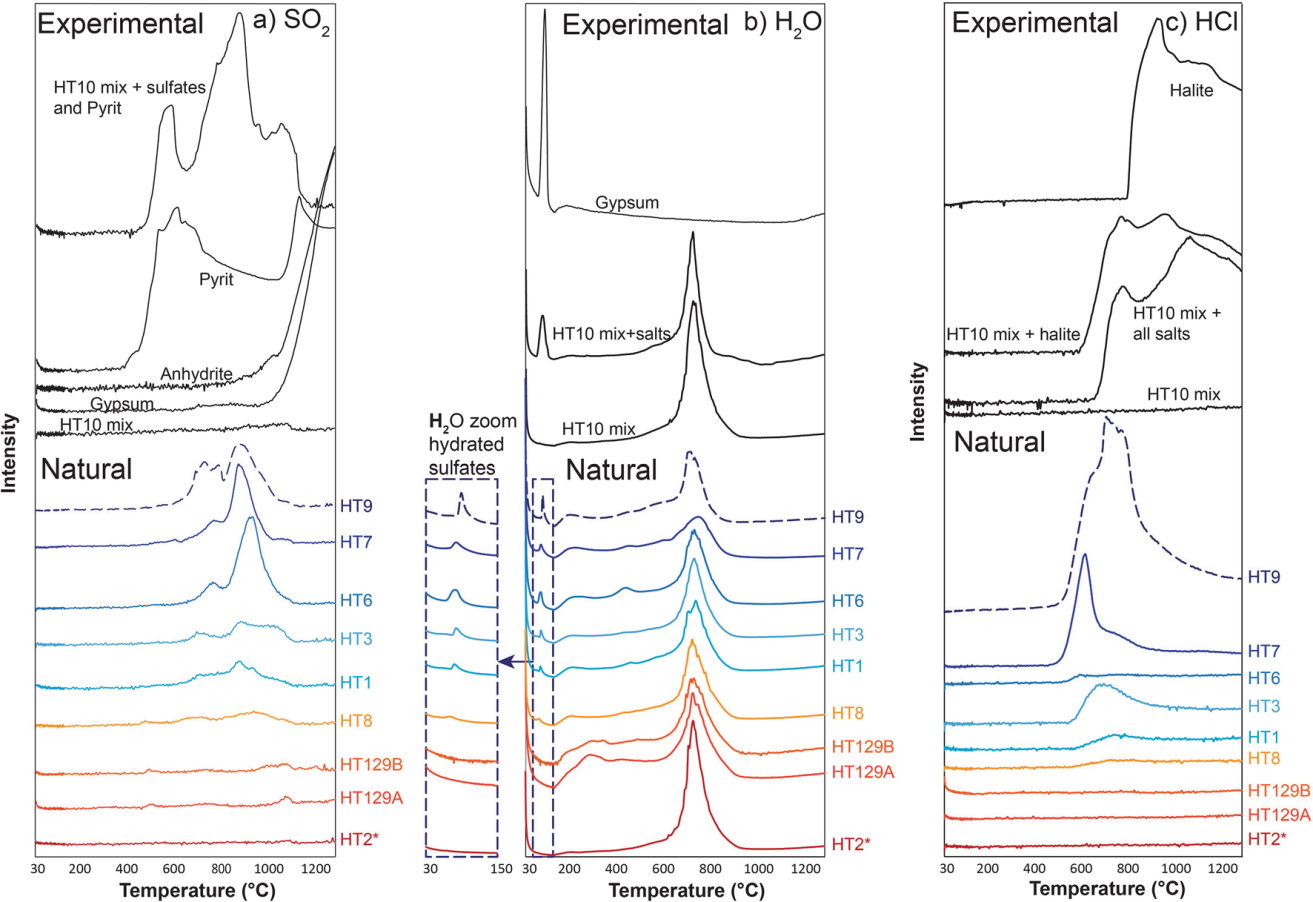
Signal of the evolved gas analysis (EGA) signal for natural samples (colourized curves) and preparations of pure glass, pure salts and sulfides and mix of these (black curves). (**a**) SO_2_ signal during thermal heating at 30 K/min. (**b**) H_2_O signal with gypsum-related peak at low temperature (< 100 °C) and magmatic water peak from the glass once T_g_ is crossed. Small signal between these two episodes of water loss may be an artefact or some loss of meteoric (non-magmatic) water from the glass. (**c**) HCl signal during thermal heating at 30 K/min. *Sample HT2 was wet-sieved before analysis and is therefore salt-free.

Molecular H_2_O, CO_2_, HCl, SO_2_ and H_2_S are all detectable as unique spectral signatures during heating-induced breakdown of Hunga ash samples. We focus here on the SO_2_, H_2_O and HCl signals that are related to the presence of salts and sulfides. The SO_2_ signal for ash samples is distinct from that of pure anhydrite or gypsum, but similar to a mixture of clean, washed Hunga glassy ash (HT10) with manually added Ca-sulfate and pyrite (Fig. 3a). This mixture has a main SO_2_ release detected between 600 and 1100 °C. The maximum SO_2_ signal in natural ash samples is highly variable and correlates with the low-temperature H_2_O peak (Fig. 3b), which is related to the presence of gypsum (Fig. 3b) and/or bassanite (2CaSO_4_·H_2_O), which shows similar behavior in thermal analysis^16^. The main H_2_O peak at ~ 700 °C is related to degassing of magmatic water from ash particles (Fig. 3b).

**Figure 4:**
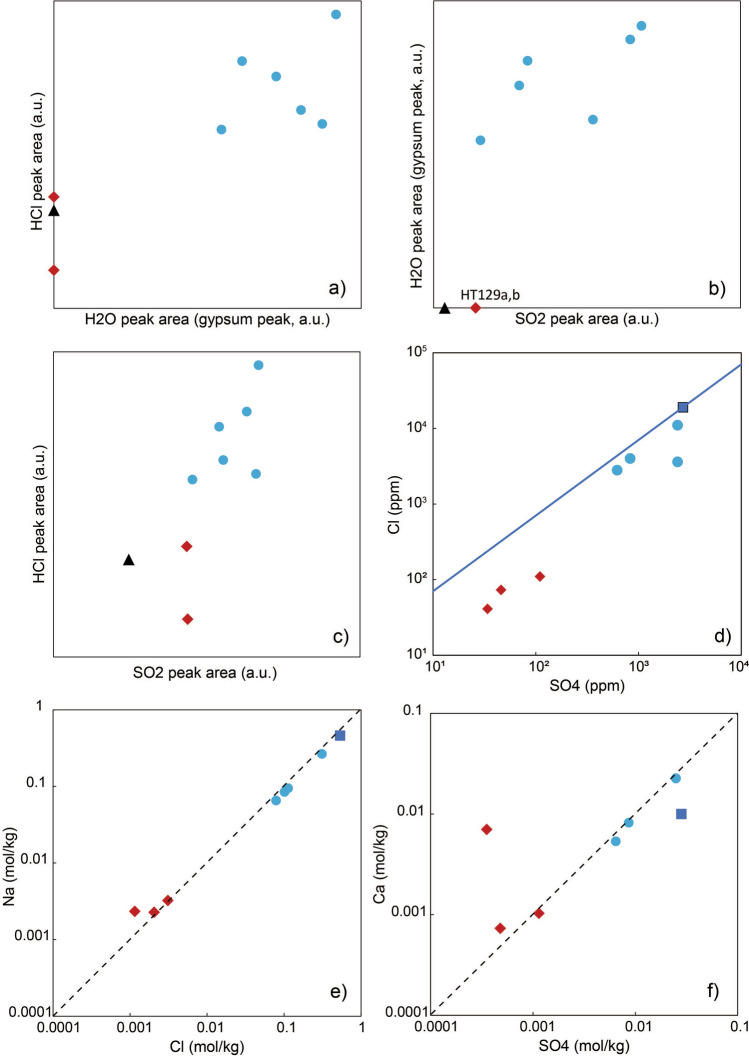
Comparison of EGA and leaching results. (**a**–**c**) Comparison of signal areas for SO_2_, HCl and low temperature H_2_O from the EGA analysis. (**d**) Results from ion chromatography with the Cl and SO_4_ concentrations. The blue line corresponds to a seawater-ash mixing line. (**e**,**f**) Molar concentrations showing the 1:1 stoichiometric relationship (black dashed line) between Na-Cl and Ca-SO_4_. Blue circles and red diamonds are Group 1 and Group 2 samples, respectively. The black triangle corresponds to sample HT2 that was wet-sieved before analysis and is hence salt-free. The blue square corresponds to the seawater concentrations.

The HCl signal in natural samples appears at around 530 °C, up to the maximum temperature of 1300 °C, and shows a distinct peak at 600–800 °C (Fig. 3c). This signal resembles that of a mixture of glass (HT10) and manually added halite, with formation of an HCl spectral signature at a temperature lower than the melting point of pure halite (~ 800 °C; Fig. 3c). Together, these data imply that the HCl, SO_2_ and low-T H_2_O are related to the presence of halite, Ca-sulfate (gypsum and/or bassanite, and possibly anhydrite) and Fe-sulfides, consistent with EDX analysis. We observe overall correlations between calculated areas of HCl, low-T H_2_O and SO_2_ signals among samples, which imply similar relative proportions of halite and Ca-sulfates (Figs. 3 and 4). This method also allows us to discriminate between salt-rich (Group 1) and salt-poor (Group 2) samples in which the signal for SO_2_, low-T H_2_O and HCl is weak or absent (Figs. 3 and 4).

The lower temperatures for the decomposition of Ca-sulfates (Fig. 3a) and melting of halite (Fig. 3c) observed in the presence of glass (HT10), as compared to pure components, are caused by the partial pressure of magmatic water released by ash particles above the glass transition temperature of ~ 535–584 °C^17^.

### Leaching

Molar concentrations of Ca-SO_4_ and Na-Cl show a 1:1 stoichiometric ratio (Fig. 4e,f) indicating dissolution of NaCl (halite) and CaSO_4_ (gypsum/bassanite ± anhydrite) from the leachates^18,19^, as also seen in EDX and EGA analyses. The amount of Cl and SO_4_ measured by ion chromatography correlates with the evolved gas analysis data for HCl and SO_2_, respectively (Fig. 4). Ion concentrations also allow us to distinguish between Group 1 (salt-rich) and Group 2 (salt-poor) samples. In particular, salt-poor samples from Group 2 (HT118, 129A and B) are characterized by low values of Cl, SO_4_, Br, Mn, Ca, K, Mg and Na, and high values of F (only in HT118, possibly due to the presence of carbonates in this sample), nitrate, Fe and Si compared to salt-rich samples of Group 1 (Table 1).

**Table 1:** Ion concentrations (in ppm) obtained from the leaching procedure for the two groups of natural ash samples.

Type	Sample name	Cl	F	SO_4_	NO_3_	Br	Fe	Mn	Ca	K	Mg	Na	Si
Group 1	HT9	11,000	< 2.0	2400	< 4.0	30	< 0.2	4.63	908	120	511	6090	2.78
	HT1	4000	< 2.0	830	< 4.0	7.8	0.62	0.92	328	55.9	163	2170	4.84
	HT6	3600	< 2.0	2400	< 4.0	12	0.53	2.97	901	95.6	179	1940	6.31
	HT8	2800	< 2.0	620	< 4.0	9.8	1.31	1.26	214	47.4	110	1500	5.14
Group 2	HT129A	110	< 2.0	110	84	< 2.0	9.94	0.24	41.2	30.8	20.1	74.4	17.3
	HT129B	73	< 2.0	46	43	< 2.0	8.35	0.154	29.2	18.8	11.5	52.3	12.9
	HT118	41	3.2	34	5.6	< 2.0	10.3	0.211	281	21.3	47.3	53.7	18.5

## Discussion

**Figure 5:**
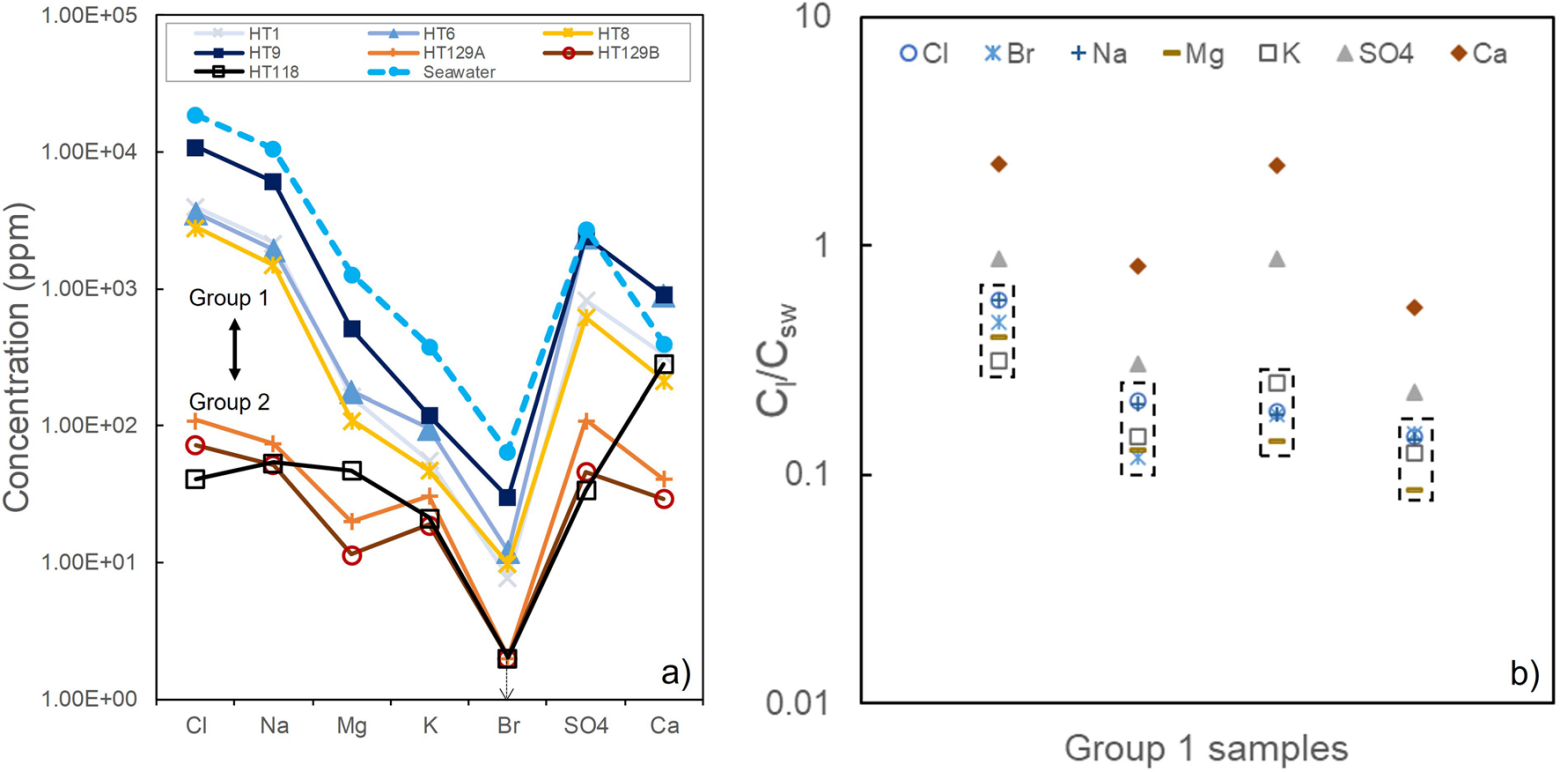
(**a**) Proportions of ion concentrations in the leachates. Only ions that are highly concentrated in seawater (values shown as blue dashed line) are included here. (**b**) Ratio of concentrations of the leachates C_l_ normalised to seawater concentrations C_sw_ for the Group 1 samples.

The highly explosive submarine eruption of andesitic magma interacting with seawater during the 15 January 2022 episode caused efficient fragmentation and ash formation, seawater boiling and extensive sea salt precipitation. Seawater is the main source of the salts observed in the deposits (halite, Ca-sulfates and bromides), although some of these may also be of magmatic origin via a process of SO_2_ (± HCl) uptake^20^. Ash samples from Group 1 all show similar variations in the concentrations and proportions of ions relative to those found in seawater (Fig. 5a; Table1). Thus, efficient mixing and transfer of elements between seawater and ash was the dominant mechanism generating the observed salt precipitates^9^. The amount of halite in the leachate HT9 is among the maximum values observed, and is only greater in data for previous Surtseyan activity at Hunga volcano from the 2014–2015 eruption^10^. Absolute values of concentrations approach those of seawater (Figs. 4 and 5). We conclude that salts observed in the deposits are derived dominantly from seawater evaporation and sea salt formation following magma-seawater interaction.

Although a large amount of these salts was deposited on land, physically bound to ash from the plume, it is also likely that a large volume of ash-free salts and aerosols remained in the atmosphere after the eruption. A previous study questioned the origin of a hazy substance visible at the top of the plume^3^**.** Similar haze is associated with littoral eruptions at Kilauea volcano (Hawaii) and is attributed to sea salts^6^. It was also recently suggested that bromine and chlorine may have been injected in the stratosphere in substantial amounts by the Hunga plume^21^.

Large amounts of sea salts transferred via volcanic plumes into much higher levels of the atmosphere than normal during such magma-seawater eruptions may cause long-term atmospheric and weather changes^6^. Dehalogenation of sea-salt aerosols, which usually originate from wave breaking and surface bubbles, is believed to be the largest source of Cl and Br gases in the troposphere^11^. During the 15 January 2022 eruption, dichlorination and debromination of aerosols carried by the volcanic plume may have released Cl and Br into the stratosphere and mesosphere. Such halogens reaching high altitudes may lead to destruction of ozone and affect the global radiation budget^11,22^. In addition, large quantities of HCl formed via seawater boiling and/or reheating of NaCl (Fig. 3) by hot pyroclasts or interaction with sulfuric acid^23^ may also have implications for atmospheric ozone^24^.

**Figure 6:**
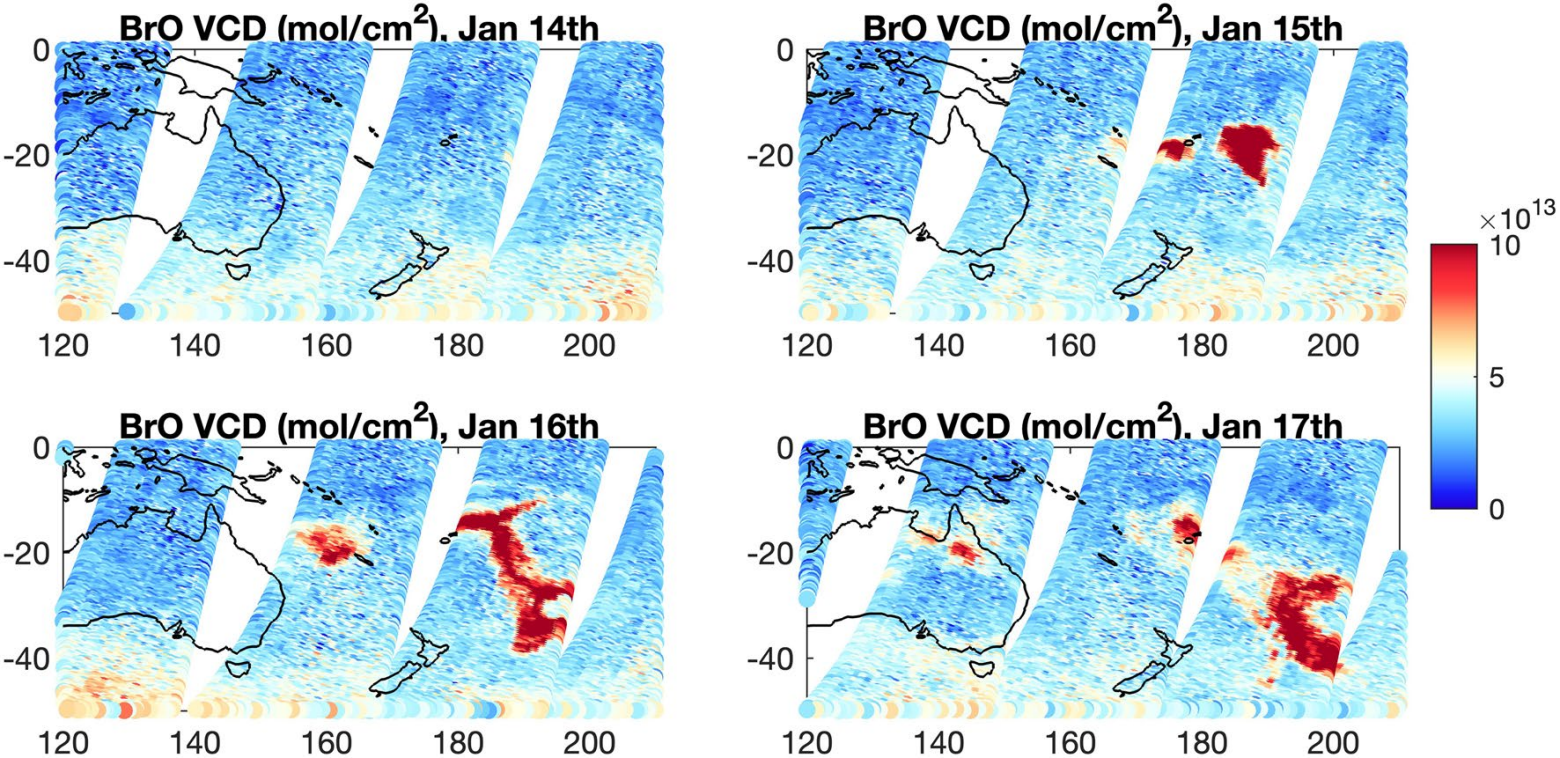
Vertical column density of bromine monoxide (BrO) from the Global Ozone Monitoring Experiment-2 (GOME-2) onboard the Meteorological Operational Satellite-C (Metop-C) for January 14th to 17th 2022.

Enrichments in HCl in the stratosphere were confirmed but these are comparable to that observed in previous, non-submarine volcanic activity^25^. Observations of total BrO columns from GOME-2 onboard the Metop-C satellite show two distinct signals of enrichment (Fig. 6). While the eastern signal is showing tropospheric BrO loading^26^, the somewhat smaller western signal is of stratospheric origin, coinciding with the stratospheric trajectory of the volcanic plume and location of H_2_O enrichments in the days following the eruption^25^. This, together with the Br/Na ratios in our leachates that are on average lower (0.0053) than in seawater (0.0065), suggests some release of Br to the atmosphere via debromination. The stratospheric plume was also associated with ozone depletion^21^; however, it is not clear how much of this signal is driven by halogen loading.

We conclude that there was a larger than usual injection of chlorides and bromides (partly transformed to HCl and BrO) to the stratosphere and mesosphere due to the submarine nature of the eruption. However, this effect was largely compensated for by the efficient binding between sea salts and ash particles in aggregates^27^, causing deposition of these salts in conjunction with ash in the sea or on land. The 15 January 2022 Hunga eruption contrasts with littoral eruptions, where lava flows enter the sea and generate salt-rich steam clouds that are mostly ash-free, and where salt production and transfer to the atmosphere is millions of times greater than for average sea surface processes^6^.

The ratio of concentrations for chloride- and bromide-bearing ions (Na, Cl, Br, Mg and K) relative to seawater in the leachates of Group 1 ranges between 0.08 to 0.58 (Fig. 5b). If all ions were transferred from seawater to the ash during complete evaporation, these values correspond to an initial seawater/magma ratio. The different ratios across Group 1 samples may be caused by variable mixing within the plume. This range of seawater relative to magma corresponds to typical values expected with high efficiency of conversion of thermal energy to mechanical work, used as a proxy for explosive energy of shallow submarine eruptions^28^. However, these values may be a minimum estimate for a seawater/magma ratio due to (i) loss of some chlorides and bromides during dehalogenation, (ii) moderate interaction with rainwater in the days after deposition (Fig. 1) and (iii) a fraction of salts formed via seawater evaporation likely released in the atmosphere and not incorporated into ash aggregates.

SO_2_ and sulfate aerosols injected in the troposphere, stratosphere and mesosphere may contribute to climate change^3^. Our concentrations of Ca and SO_4_ cannot be explained by seawater and ash mixing only. The ratio of Ca_sample_/Ca_seawater_ and SO_4sample_/SO_4seawater_ both vary in a similar pattern across samples from Group 1, but with a distinct trend compared to other ions and with significantly higher values (Fig. 5b; Table 1). This implies additional Ca-sulfate formation via SO_2_ scavenging and Ca diffusion from the volcanic glass at the surface of the ash particles^20,29^. As for chlorides and bromides, efficient trapping in ash aggregates of Ca-sulfates, both originating from seawater boiling and from scavenging, promoted deposition and limited their impact on the atmosphere. In contrast to the size of the eruption, only small masses of SO_2_ were detected in the stratosphere as a result of the 15 January plume (~ 0.4–0.5 Tg^30^). The discrepancy between the SO_2_ measured by satellites and values expected from degassing (~ 24 Tg^31^) may be explained by gas scavenging of SO_2_ and formation of Ca-sulfates, and the deposition of such CaSO_4_ within ash aggregates or release as free salt particles in the atmosphere. In addition, a large amount of SO_2_ may have been released to the sea via passive degassing prior to the eruption or with ejecta in submarine gravity currents.

Combining seawater concentrations and estimates of water vapor in the atmosphere (~ 50–140 Tg^21,22^) and assuming that water vapor comes dominantly from seawater injection, we can calculate a range for the total budget of ions injected in the atmosphere via seawater evaporation. This yields 0.95–2.66 Tg Cl, 0.14–0.38 Tg SO_4_ and 0.003–0.01 Tg Br (possibly partitioned both as solid particles and gas), with upper values for Cl and Br comparable to total annual gas emissions from global volcanic activity^32^. Combining the same range of water vapor estimates with our calculated seawater/magma ratio, we estimate the mass of magma injected in the atmosphere, which yields 86–1750 Tg. These values are much lower than the mass of magma calculated from the volume change related to caldera collapse of 7.9 km^3^ Dense Rock Equivalent^33^, i.e., 22,000 Tg, however much of this volume appears to have been deposited in the form of pyroclastic density currents on the sea floor. Estimates of fall tephra from regression of measured values suggests a volume of ~ 1.5 km^3^ (Cronin et al., pers comm.), which corresponds to ~ 0.6 km^3^ DRE or ~ 1700 Tg.

A high concentration of salts in the volcanic plume increases the stability of aggregates^27^ with implications for ash residence time in the atmosphere, plume dispersal, depositional location and related hazards. Efficient cementation of ash aggregates by sea salts has been discussed for phreatomagmatic eruptions at Stromboli^34^ and for the 2014–2015 Surtseyan activity at Hunga volcano^10^. The exact role of salts on ash deposition during the 15 January 2022 eruption requires further investigation since aggregation can either promote or delay sedimentation depending on porosity and aggregate-to-core size ratio^35^. Salts may also have consequences for volcanic lightning processes, since the combination of seawater boiling and extensive salt formation leads to the formation of electrically charged aerosols^36–38^. The exact influence of salt formation on volcanic lightning during the Hunga eruption is unknown, and along with the inferred high water/ice content could explain the extreme density of lightning reported for this eruption^39^.

Data from three independent methods confirm the presence of a large amount of sea salts in the tephra deposits from the 15 January 2022 eruption of Hunga volcano. Evolved gas analysis (EGA) is a novel approach that provides semi-quantitative data on the concentration of soluble salts in volcanic ash, providing a complementary approach to leaching studies. Samples from Group 2 that were exposed to rain for several months are characterized by a low amount of salt that reflects post-depositional dissolution of salts exposed to rainwater, highlighting the importance of rapid sampling in these types of deposits. Samples of Group 1 that were rapidly collected after the eruption show the highest salt content. Many chlorides in Group 1 samples preserved their crystal morphologies (Fig. 2) in contrast to deposits from the 2014–2015 Hunga volcano eruption that were dominated by smooth morphologies^10^ resembling dissolution textures^5^. This suggests that primary dissolution in the plume was limited during the 15 January 2022 eruption, despite possible interaction with ice. The high pristine salt concentration in the ash deposits is evidence for large-scale injection of sea salts up to the mesosphere. A large part of these salts did not remain in the atmosphere and was deposited in aggregates, likely due to efficient binding between salts and ash particles. The balance between free salts remaining in the atmosphere and those that were deposited within ash aggregates on land or in the sea is a key control on the atmospheric salt budget and related climatic impacts during breaching submarine eruptions.

Satellite measurements of gases in volcanic plumes are currently used as a proxy for rapidly constraining eruption size and climatic impact, and are available much earlier than ground-based measurements, especially in remote areas such as oceanic islands. It was proposed that the main climatic effect of the 15 January 2022 eruption of Hunga volcano was the unprecedented volume of observed H_2_O injection, whereas SO_2_ and HCl were discussed as unexceptional^25^. Our study shows that several complex processes due to the submarine environment should be considered to interpret satellite measurements. It is vital to consider for calculations of the volatile budget in the atmosphere, that the primary source for HCl, BrO, H_2_O, and to a lower degree SO_4_, is seawater and not volcanic volatiles for this type of eruption. For all the produced Cl- Br- and SO_2_/SO_4_-bearing species, partitioning between the sea, ash aggregates and free volatiles or salt particles released in the atmosphere needs to be taken into account in future studies. Thus, we recommend that in order to determine the magnitudes of submarine eruptions and their impacts on the atmosphere, a multi-faceted approach is needed. This would combine rapid sampling and analysis of pristine tephra with remote sensing techniques of the eruption plume for a wide range of volatile elements and salt particles.

## Methods

### Sampling

Samples from Group 1 were taken in the first two weeks after the eruption. The first set (HT1, HT2, HT3 and HT9) was collected by military personnel from piles of ash swept from the Fu’uamoto airport runway a week after the eruption (22 January 2022), in three clean polyethelene containers (ca. 80 km SSE of Hunga), on Tongatapu Island. Fall is mapped at this location as 1.8–2 cm-thick. HT9 was sampled at the office of Tonga Geoscience Services in Nuku’alofa (ca. 67.9 km S from Hunga) on Tongatapu Island, under phone instruction from SJC and brushed into sealed zip-loc plastic bags. HT9 is coarse to fine, poorly sorted ash, including very fine ash and lapilli up to 4 mm. Fall is mapped at this location at 2.7–3 cm thick. HT9 is considered the most pristine sample due to the sampling location, the large size of the bulk sample (~ 20 kg), and low rain in the area prior to sampling. Sample HT2 was wet sieved prior to analysis and is therefore a salt-free sample. All samples are fine grained and uncontaminated. Two weeks later on 28 January, another set of samples from Group 1 was collected (HT6, HT7 and HT8) (Fig. 1). Virtually no rain occurred in the two weeks after the eruption, except for isolated showers of light rain, but no heavy or persistent rainfall was observed. HT6 was collected on Nomuka Island (ca. 69.5 km ENE from Hunga), it is a fine ash, moderately sorted and contains some foreign material including rock, gravel, and calcareous sand. The rest of the samples from Group 1 did not exhibit this biological component. HT7 was taken on Fonoifua island (ca. 84.2 km ENE from Hunga) and consists of moist and moderately sorted fine ash. HT8 was collected on Tungua island (ca. 88.6 km NE from Hunga) and presents the same characteristics as HT7.

The last set of samples (Group 2), which includes HT118 and HT129A-B, was taken carefully attempting to sub-sample depth slices representing the stratigraphy of the deposit. It was sampled in April, from new sites NE from Hunga. However, the month of February saw increasing rainfall, which remained constant in March, about 159 mm throughout, and rarely exceeding 298 mm (Fig. 1)^40^. HT118 was sampled on the 18 April on Nomuka-iki island (ca. 68 km ENE from Hunga) and corresponds to the latest stage post-tsunami ash, collected on tsunami deposits near the coast. It contains abundant carbonate seashells due to post-emplacement contamination. HT129A-B were sampled on 19 April, ~ 63.4 km E from Hunga, from a second and last fall, before and after tsunami deposits respectively (basal and top ash). The rainfall and leaching may have also strongly altered the deposit stratigraphy, e.g., by washing fines to lower levels and concentrating particles at the upper deposits surface.

### SEM analysis

Back-scattered electron (BSE) images and energy-dispersive X-ray spectroscopy (EDX) were combined using a HITACHI SU 5000 Schottky FE-SEM at LMU, in order to determine the nature, size and morphology of salts and to semi-quantitatively assess the salt coverage on ash particles^29^. Salt coverage was estimated by binarization of the chemical maps of Cl and S followed by a 1-pixel erosion and dilation step to remove noise.

### Thermal analysis

We performed Evolved Gas Analysis (EGA) on a Mettler-Toledo TGA–DSC 3 + attached to a Pfeiffer Vacuum GSD 320 gas mass spectrometer^41^, allowing us to determine the nature of meteoric, magmatic and salt-related volatile species during thermal treatment. Samples were heated in Nitrogen at a rate of 5 °C/min from 30 to 150 °C for dehydration purposes and then at a rate of 30 °C/min from 150 °C to 1300 °C passing through the glass transition temperature range (T_g_). We speculate T_g_ values of ~ 535–585 °C although these values correspond to glassy samples from the 2014–2015 eruption^17^. During heating, the relative amount of different volatile species (H_2_O, CO_2_, SO_2_, HCl, and H_2_S, which are extracted from mass-to-charge ratios of 18, 44, 64, 36, and 34, respectively) were recorded by the gas mass spectrometer. Natural samples collected for the analysis were bulk ash material or correspond to a given ash fraction. Particles were unwashed and dried at 40 °C before analysis. We compared the signals for natural samples with (i) a mixture of salt-free dense glassy particles, white and dark pumice, (ii) pure halite, anhydrite, gypsum, calcite and pyrit, (iii) mix between pure magmatic particles from (i) and components from (ii). This mixing and progressive heating of the samples allowed us to separate the signals of (i) meteoric volatiles formed by rehydration (volatile loss at temperatures below T_g_), (ii) magmatic volatile content (volatile loss above T_g_ in the glassy, salt-free samples) and (iii) salt decomposition/melting reactions. The signals for each natural sample were corrected for the initial sample mass (which ranges between 16 and 29 mg).

### Leaching procedure

Leachates of the same sample suite analysed for BSE/EDX and EGA analysis were prepared with dilution ratios of 1:20. Ash samples were mixed with deionized water for one hour and then filtrated at less than 2 µm on a Cellulose filter. The leachates were analysed at LMU by ion chromatography to quantify the content of anions (Cl, Br, F, nitrate, SO_4_), whereas the cation concentrations were determined by Inductively Coupled Plasma Optical-Emission Spectroscopy (ICP-OES).

### Satellite measurements

We use BrO vertical column density from the Global Ozone Monitoring Experiment-2 (GOME-2) onboard the Meteorological Operational Satellite-C (Metop-C) for the time period January 14th 2022 to January 17th 2022. The data is based on the GOME Data Processor (GDP) operational algorithm version 4.9, which uses an optimized Differential Optical Absorption Spectroscopy (DOAS) algorithm to determine the trace gas slant columns followed by air mass factor conversions to generate vertical columns.

## Data Availability

All data generated or analysed during this study are included in this published article and in a data repository (https://doi.org/10.5880/fidgeo.2023.014;^42^).
